# Pro-tumorigenic non-pump function of sodium iodide symporter: A reimagined Trojan horse?

**DOI:** 10.18632/oncotarget.26596

**Published:** 2019-01-22

**Authors:** Fang Feng, Lamis Yehia, Charis Eng

**Affiliations:** Charis Eng: Genomic Medicine Institute, Lerner Research Institute, Cleveland Clinic, Cleveland, OH, USA; Taussig Cancer Institute, Cleveland Clinic Foundation, Cleveland, OH, USA; Department of Genetics and Genome Sciences, Case Western Reserve University School of Medicine, Cleveland, OH, USA; Germline High Risk Cancer Focus Group, CASE Comprehensive Cancer Center, Case Western Reserve University, Cleveland, OH, USA

**Keywords:** thyroid cancer, PTEN, sodium iodide symporter, migration, Cowden syndrome

“*The real voyage of discovery consists not in seeking new landscapes, but in having new eyes.*” – Marcel Proust

The sodium iodide symporter (NIS), encoded by the *SLC5A5* gene (solute carrier family 5, member 5), is a transmembrane glycoprotein expressed at high levels on the plasma membrane of thyroid follicular epithelial cells, to mediate iodide uptake from the bloodstream for thyroid hormone biosynthesis. Although *SLC5A5* was cloned in 1996, the ability of the thyroid to accumulate iodide was first described by Baumann in 1896. Several decades later, radioiodide-131 (^131^I) was first utilized to treat hyperthyroidism in 1939, and then administered to treat thyroid cancer in 1946. Thus far, ^131^I treatment is the most effective targeted internal radiation therapy, making NIS a crucial molecule in studies of thyroid diseases. Interestingly, NIS also exists physiologically in extra-thyroidal organs such as the salivary gland, stomach, intestine, renal tubules and the lactating breast. Similar to thyroid cells, NIS localizes to the cell membrane in these extra-thyroidal tissues and mediates iodide uptake from the gastrointestinal tract, excretion by the kidneys and secretion into milk within the breasts. Hence, NIS classically plays an important role as a transmembrane iodide pump [1].

The idea that NIS may have pro-tumorigenic activity emanates from the observation that NIS is elevated in multiple non-thyroidal cancers but is absent in their normal tissue counterparts. Intriguingly, NIS is located predominantly intracellularly within these tumors, with low or absent iodide pump function. The most frequently investigated cancers are breast cancers, 70–80% of which have been reported to have high levels of intracellular NIS protein [2]. Importantly, the increased NIS protein levels have been recently reported to be associated with poor prognosis and chemotherapy resistance in breast cancer [3], and aggressive behavior in prostate cancer [4]. Our most recent work revealed that cytoplasmic NIS results in an increased cellular migration phenotype in thyroid cancer cells [5].

The interaction of NIS with other relevant proteins provides further evidence of its non-pump functions. Earlier studies indicated that the secondary-structure of NIS contains a putative density-95/discs large/zona occludens-1 (PDZ) class 1 target motif (T/S-X-V/L) at the COOH-terminus, endowing it with the ability to interact with other proteins through PDZ protein-protein interactions. Our study and others’ reveal that NIS can interact with leukemia associated RhoA guanine exchange factor (LARG) to activate RhoA in thyroid and non-thyroidal cancer cells [5, 6]. Other studies have shown that NIS can interact with pituitary tumor transforming gene (PTTG) binding factor (PBF) in the cytoplasm of COS-7 monkey kidney and FRTL-5 rat thyroid cell lines [7]. Hence, the NIS interactome warrants further investigation as it represents a germane opportunity to further explore non-canonical functions of this protein.

Interestingly, both thyroid cancer and breast cancer represent the most frequently observed component cancers in Cowden syndrome (CS), an inherited overgrowth and cancer predisposition syndrome, classically associated with germline *PTEN* mutations. We observed that NIS crosstalks with the PTEN-PI3K/AKT/mTOR signaling pathway, presenting its pro-tumorigenic function independent of its iodide pump activity in thyroid cancer cell models [5]. As a tumor suppressor gene, *PTEN* is somatically mutated in multiple cancer types and is considered as one of the most frequently somatically mutated genes in human cancers. Hence, PTEN loss may have downstream implications on the protein level and localization of NIS in tissues where the latter is expressed. Thus, we speculate that this crosstalk may be generalizable in multiple non-thyroidal cancers as well.

Importantly, since NIS forms the basis of radioiodide treatment in thyroid cancer, the existence of non-pump functions in thyroid cells may have profound clinical implications. Supportively, although radioiodide uptake and NIS mRNA expression are generally decreased in differentiated thyroid cancer (DTC) compared to normal thyroid tissue, several groups have reported elevated intracellular NIS protein levels in DTC [8]. An important finding from our study is demonstrating that *PTEN* loss results in increased NIS protein levels. We believe that mis-localization of NIS in the cytoplasm endows it with the ability to interact with other cytoplasmic proteins. This, in turn, promotes its non-pump function in thyroid cancers, and increased intracellular NIS stability through these protein-protein interactions. However, it remains vital to elucidate the factor(s) affecting NIS subcellular localization. Several studies have reported that, as a glycoprotein, glycosylation levels play important roles in NIS transmembrane localization [9]. Consistently, agents which can enhance protein glycosylation can also enhance NIS cell membrane trafficking. Our data indicate that PTEN signaling impacts the glycosylating enzyme DPAGT1 to then regulate NIS glycosylation, providing one possible mechanism.

Importantly, following the successful utilization of radioiodide for the treatment of DTC, some studies have investigated the potential application of NIS gene therapy in the treatment of iodide refractory thyroid cancers and some non-thyroidal cancers [10]. Increased iodide uptake in cancer cells after NIS transfection was indeed observed, however, the therapeutic effect remains inconclusive. If our observations are correct, then therapeutic strategies should go beyond only increasing NIS protein levels towards promoting NIS to express specifically on the plasma membrane. We and others have shown that PI3K/AKT/mTOR inhibitors can increase plasma membrane NIS. Our data further elucidate that these pharmacologic agents also decrease intracellular NIS, indicating their possible utility in the treatment of thyroid cancers, from the point of clearance of the pro-tumorigenic non-pump-functional NIS.

More and more, the realization that not just proper levels of expression of a protein are required for appropriate function, but also proper intracellular localization. One classical example is PTEN, first believed to be an exclusively cytoplasmic protein, and now known to exist in the nucleus, contributing to major functions such as genomic stability, cell cycle regulation, DNA double-strand break repair, chromatin remodeling, and ribosome biogenesis. The other classical tumor suppressor p53, normally functioning as a transcription factor in the nucleus, can accumulate in the cytoplasm of some cancer cells. Similarly, the oncoprotein epidermal growth factor receptor (EGFR), classically localizing on the plasma membrane, has been observed to traffic into the nucleus, contributing to a cancer phenotype. Therefore, it will be prudent to consider the spatial distribution of NIS and other proteins to circumvent possible unsuspected pro-tumorigenic signals.

“…*location, location, location*.” – Lord Harold Samuel (real estate tycoon)
